# State-of-the-art in automated image-based species identification systems

**DOI:** 10.3389/fcimb.2026.1818023

**Published:** 2026-10-20

**Authors:** Lukas Weber, Mohammedaman Mama Hussen, Balaji Veeraraghavan, Eline Meijer, Yukino Gütlin, Alejandro Guerrero-López, Sarah Brüningk, Oliver Nolte, Catherine Jutzeler, Adrian Egli

**Affiliations:** 1 Institute of Medical Microbiology, University of Zurich, Zurich, Switzerland; 2 Goba Referral Hospital, Madda Walabu University, Robe, Ethiopia; 3 Department of Clinical Microbiology, Christian Medical College and Hospital Vellore, Vellore, India; 4 Department of Radiation Oncology, Inselspital, Bern University Hospital and University of Bern, Bern, Switzerland; 5 Department of Digital Medicine, University of Bern, Bern, Switzerland; 6 Department of Health Sciences and Technologies, ETH Zurich, Zurich, Switzerland

**Keywords:** artificial intelligence, convolutional neural networks, culture morphology, image analysis, species identification, Siamese networks, object detectors, Vision Transformers

## Abstract

Accurate and rapid species-level identification of bacteria and fungi from culture plates are longstanding challenges in diagnostic microbiology. Visual assessment of colony morphology lacks sufficient specificity for definitive identification, while biochemical profiling is often too slow for routine clinical use. Matrix assisted laser desorption ionization - time of flight mass spectrometry (MALDI-TOF MS) has largely replaced biochemical identification in high-income settings because of its speed and accuracy; however, its cost and infrastructure requirements limit accessibility in most low resource settings. Artificial intelligence (AI), particularly convolutional neural networks (CNNs), offers a promising alternative by enabling growth detection and presumptive identification direct from digitalized macroscopic images of culture plates. This review summarizes key aspects of visual identification from agar cultures and recent advances in image-based microbial analysis using CNNs, Siamese networks, object detectors, and Vision Transformers. Automated growth detection and whole-plate interpretation achieved sensitivities of 99.8–100%, specificities of approximately 63–90%, and within-institution AUCs of ≥0.98, although cross-institution performance decreased to ≥0.90; reported workflow benefits included 3–5-hour reductions in time to result and up to 80% less hands-on review of negative cultures. Species-level bacterial classification ranged from a mean sensitivity of 66.2% in an early 18-species model to 90.6% top-1 and 97.6% top-3 accuracy with the contextual DeepColony framework. Fungal models achieved a mean accuracy of 89.7% with FungID and an AUROC of 92.7% with smartphone-based MoldVision, whose F1 score increased from 38.8% during days 1–2 to 92.1% during days 3–5, highlighting the influence of colony maturity, dataset composition, and validation setting. We further discuss the integration of AI image analysis in commercial laboratory automation systems, including Copan WASPLab™, BD Kiestra™ MLA, APAS™ Independence by Clever Culture Systems, as well as the potential of smartphone-based tools in resource-limited settings. Overall, AI-based image analysis can support high-sensitivity screening, workflow triage, colony enumeration, and presumptive identification, but robust external validation, standardized imaging and reporting, regulatory approval, and continued expert oversight remain essential.

## Introduction

A critical step in clinical microbiology is culturing patient specimens on agar plates followed by identification of grown pathogens. This process is time-consuming, requiring 18 to 24 hours for culturing of most mesophilic bacteria, up to 96 hours for anaerobes, or within three to four days for *Cutibacterium* spp., and up to several weeks for slow growing organisms such as *Mycobacteria tuberculosis*. Interpretation of growth in clinical context and processing of candidate microorganisms for identification requires skilled and highly trained personnel. Modern methods, such as Matrix assisted Laser Desorption Ionization - Time of Flight mass spectrometry (MALDI-TOF MS), have accelerated identification, in comparison to biochemical identification profiling, once a pure colony is obtained (Mellmann et al., 2008; Arbefeville et al., 2024). The initial assessment of colony morphology on agar plates remains critical and skilful. Lab technicians and microbiologist first screen, if there is visible growth. If colonies are detected, the morphology, e.g., size, shape, colour, haemolysis, etc. allows a first categorization but often provides only a presumptive identification, narrowing possibilities but rarely pinpointing the correct species. As example, a crater-like (autolysis), grey-greenish, shiny colony with alpha haemolysis is likely a *Streptococcus pneumoniae*. Morphological characteristics are dependent on growth conditions (Sousa et al., 2013) and subtle visual differences are difficult to interpret and require expertise (Huang and Wu, 2018; Lucking et al., 2021). Moreover, reading plates is tedious and subject to human fatigue and variability.

Laboratory automation is now bringing the *digital age* to culture plates. Digital imaging creates an opportunity for artificial intelligence (AI) and its deep learning algorithms to assist in interpreting culture morphology of bacteria growing on agar plates. Direct screening for colonies (growth/no growth) is the first step, followed by species-level identification directly from digitized plate images. This approach has the potential to revolutionize workflow by providing early presumptive identifications prior to confirmatory testing with e.g., biochemical methods or MALDI-TOF MS. AI-driven image analysis in medical microbiology is best viewed as an augmentation technology: routine plate reading is a high-volume, repetitive, and operator-dependent step that is vulnerable to fatigue-related variability, yet it directly determines which colonies are worked up and how quickly actionable results reach patients. By streamlining repetitive visual tasks, AI-assisted plate reading can standardize growth detection and triage, safely auto-clear true negatives, and prioritize likely pathogens for confirmatory testing, improving efficiency, quality, turnaround times, consistency, and resilience against workforce shortages. Importantly, it does not replace trained laboratory technologists. Expert oversight remains essential for pre-analytics, context-aware interpretation, quality assurance, and exception handling. These tools may help close emerging workforce gaps as laboratories face increasing staffing pressures. Several use cases for species identification via AI have been documented in the literature (Faron et al., 2016a; Faron et al., 2016b; Cherkaoui et al., 2019; Van et al., 2019; Baker et al., 2020; Gammel et al., 2021) and will be discussed.

Most image recognition tools build on variations of convolutional neural networks (CNNs), which have shown great promise in computer vision tasks and are now being applied to agar plate images (Majchrowska et al., 2021). CNNs learn visual features, i.e., edges, textures, and patterns from raw images and classify images into categories, e.g., microbial species (**Figure 1**). In this review, we give insight to key model architectures and explore latest developments in applying AI-based techniques to microbial identification. We provide a comprehensive overview, pointing out critical aspect of implementing AI-based image analysis, and next generation developments on the verge to clinical application.

**Figure 1:**
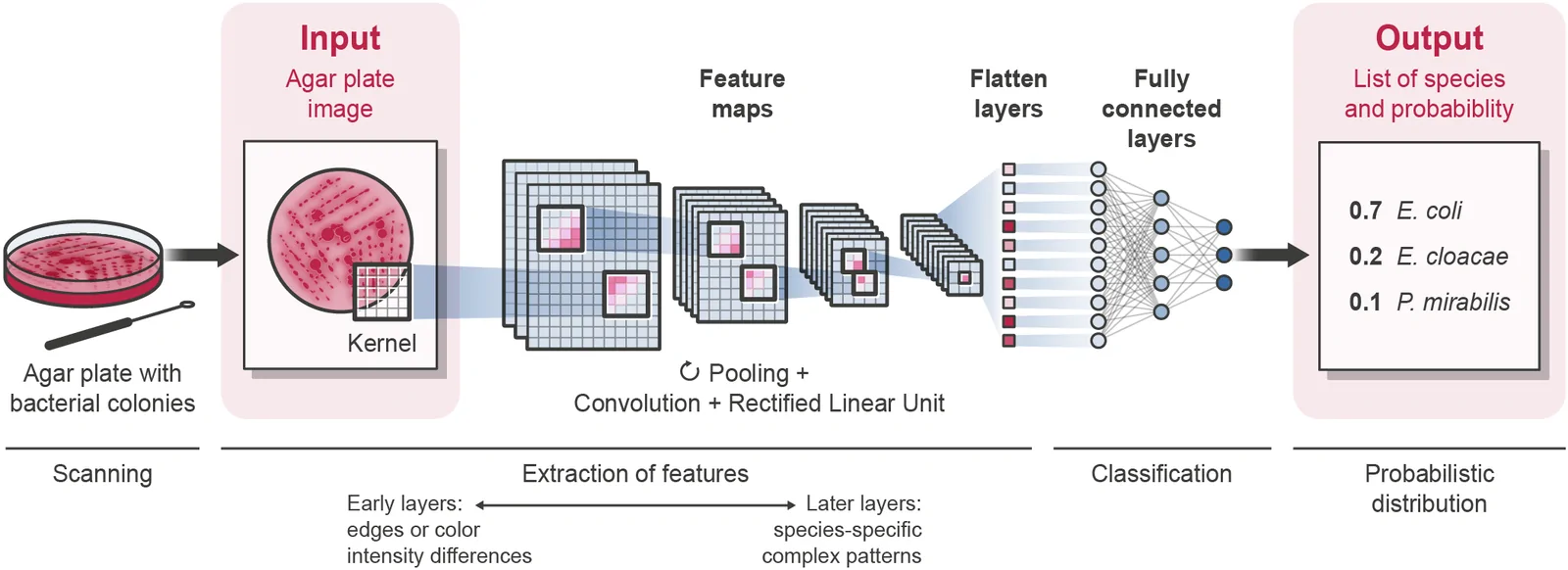
Functional aspects of a convolutional neural network (CNN) to recognize bacterial colonies growing on agar plates. The raw image (left, showing a culture plate with colonies) is passed through successive convolutional layers that extract features (middle panels). Early layers detect simple features (e.g. edges or colour intensity differences), producing feature maps, while later layers identify complex pattern characteristics of a particular species. Finally, the network’s output layer provides a classification with associated probabilities (right). The flow shows the typical recognition from an e.g. McConkey plate to an *Escherichia coli*.

## Advances in culture image assessment

Early attempts to classify colony images with machine learning date back over a decade and often relied on hand-crafted image features (Marcoux et al., 2014; Maeda et al., 2018), e.g., colour or shape metrics, combined with classifiers developed before the deep learning era, including Support Vector Machines (SVMs), Random Forests, and k-Nearest Neighbour algorithms. These had limited success due to the complexity of microbial appearances; however, these models were much easier in terms of explainability, remaining easily interpretable compared to Deep Learning methods. The advent of deep learning, which learns features directly from images, provided a breakthrough. CNNs are well-suited for image analysis (Amano et al., 2022; Wu and Gadsden, 2023) (**Supplementary File 1**). CNNs process input images through successive layers that apply convolution operations using small, trainable filters (kernels). Each convolution involves sliding a kernel across the image to compute dot products between the kernel and local image regions, thereby extracting spatial features such as edges, textures, or gradients. In practice, each layer learns multiple kernels, and nonlinear activations help transform these features into more informative representations. As signals propagate through deeper layers, the network hierarchically combines features into increasingly abstract representations, enabling detection of complex structures, such as colony morphologies (**Figure 1**). Ultimately, the network outputs a prediction, such as the most likely species. This layered feature extraction enables CNNs to handle subtle visual differences between microbial colonies far better than manual feature engineering. Large training datasets with standardized and contextualized images allow algorithms to learn and rapidly determine microbial species. A range of different deep learning tools have been developed (**Supplementary Text 1**).

First steps of image analysis in medical microbiology. Huang & Wu were among the first to utilize deep learning for colony identification, with a CNN trained to classify colonies from 18 bacterial species on 4982 images. Their model achieved an overall species-level classification accuracy of 0.967 (range 0.919 – 0.997), precision of 0.723 (0.189 - 0.975), sensitivity of 0.662 (0.238 – 1.000), and specificity of 0.983 (0.928 – 0.999). The authors envisioned the system as a pre-screening tool to rapidly narrow down identities of colonies before confirmatory tests (Huang and Wu, 2018). Around the same time, others began developing datasets of colony images to facilitate deep learning. Majchrowska et al. introduced “AGAR,” an open dataset of 18,000 plate photos, containing single and mixed cultures of five microbial species with >336,000 individual colonies annotated (Majchrowska et al., 2021). The authors demonstrated that CNN-based object detection algorithms could simultaneously localize and identify colonies using modern detectors, e.g., Faster Region-based CNN (R-CNN), a two-stage detector, and one-stage detectors achieved high performance in classifying colonies by species while pinpointing their positions (Majchrowska et al., 2022; Makrai et al., 2023). This capability is important for plates with mixed flora, allowing to identify multiple species co-existing.

Notable architectures and advances of image analysis. Most studies have employed CNN-based classifiers, often fine-tuned from architectures originally developed for general image recognition tasks. Popular examples include residual network (ResNet (He et al., 2015)) or visual geometry group (VGG (Dutta, 2019)), which have been adapted for use in microbiology (**Supplementary File 1**). In diagnostic practice, AI-based image analysis usually follows a two-step approach with (i) recognition of growth and (ii) identification of growing species.

*Recognition of growth.* AI-based algorithms can detect colony growth on images with high sensitivity (Bailey et al., 2019): A study from 2019 evaluated an algorithm analysing 1,581 urine cultures, reporting a sensitivity of 99.8% and specificity of 68.5%. Two manual-positive/automation-negative (MP/AN) cases occurred, and among 170 manual-negative/automation-positive (MN/AP) cases, 116 involved microcolonies missed by human technologists (Faron et al., 2019). A larger study from 2020 analysed 12,931 urine cultures, showing an overall sensitivity of 99.8% and specificity of 72.0%. Most MP/AN discrepancies (91.1%) were due to expert rules classifying specimens as contamination or normal flora rather than threshold issues. Of nine MP/AN cases, eight resulted from microcolonies deliberately ignored by the software, and one was due to borderline significant colony counting (Faron et al., 2020).

*Identification of bacterial species.* The next step is direct species identification. In Baker et al. the WASPLab™ Chromogenic Detection Module (CDM) software for analysing vancomycin-resistant enterococci (VRE) from chromogenic agar was compared to manual technologist readings across three laboratories. A total of 104,730 specimens were analysed, with CDM automation showing an 90.1% agreement compared to manual analysis, achieving 100% sensitivity and 89.5% specificity. Secondary review of 10,348 initially discordant results found 499 cultures missed by technologists, 1,616 borderline colour interpretations, and 8,234 cases where interfering pigmentation from sample residues or yeast affected visual assessment (Faron et al., 2016a). Another study with 676 residual vaginal-rectal swabs explored the combination of CHROMID^®^ Strepto B agar with the PhenoMATRIX^®^ AI (PM) CDM software for detecting group B streptococcus (GBS). The study compared the performance of the software to manual reading and nucleic acid amplification testing (NAAT). CHROMID^®^ combined with PM CDM showed a 100% sensitivity in identifying true-positive GBS isolates after 48 hours, outperforming CHROMID^®^ alone (90.3% sensitivity). However, CHROMID^®^plus PM CDM had lower specificity (63.0%) compared to NAAT (97.7%) and CHROMID^®^ alone (95.4%) (Baker et al., 2020). Similar examples exist for group A streptococci (Van et al., 2019), VRE (Cherkaoui et al., 2019), or methicillin resistant *Staphylococcus aureus* (Faron et al., 2016b; Gammel et al., 2021).

Other teams have explored lightweight CNN variants to support applications with limited computational resources. For example, depth-wise separable CNNs, which reduce computational cost by factorizing standard convolutions. However, those models have been employed for colony recognition with only small datasets (Mai and Ishibashi, 2021; Karthik et al., 2024). Additionally, many studies have leveraged transfer learning, in which CNNs pre-trained on large, general-purpose image datasets, e.g., ImageNet, are fine-tuned on colony image datasets. This approach has proven especially valuable in microbiology, where annotated image datasets are typically small and labour-intensive to generate (Ferrari et al., 2015; Hay and Parthasarathy, 2018; Wang et al., 2020).

Identification of fungal species. Pouris et al. developed FungID using a VGG16 model to classify fungal species on chromogenic agar. The model was trained on a dataset of 269 images representing ten fungal species, including *Aspergillus* and *Alternaria* strains. Despite the modest dataset size, the model achieved strong classification performance, with test accuracies ranging from approximately 80% to 90% for most species after 25 training epochs (Pouris et al., 2025). We have recently developed MoldVision, a smartphone-imaging and deep-learning workflow to automate mold identification from routine culture plates. We used 161 clinical isolates across *Penicillium*, *Aspergillus flavus, A. fumigatus*, *Fusarium* spp., and *Cladosporium* spp. and >4,000 top/bottom images collected over five days of growth with different growth morphologies. We trained VGG16 CNNs and benchmarked them against feature-based models and human experts. The best model (dual-head VGG16) reached a mean AUROC of 92.7% with 68.7% sensitivity, improving strongly from days 1–2 (F1 38.8%) to days 3–5 (F1 92.1%) microbiology (**Supplementary File 1**). In mature cultures it often matched or outperformed expert readers, supporting a low-cost, scalable approach for labs lacking specialized mycology expertise with data/code shared for external validation (Weber et al., 2026).

Special architecture. Beyond standard CNN classifiers, specialized architectures tackle the unique challenges of culture plates: Siamese CNNs consist of twin CNN branches that output feature embeddings for two input images, which can be compared to measure similarity. In the context of microbial identification, a Siamese network can be trained to output similar embeddings for colonies of the same species, even if they come from different plates or patients (Signoroni et al., 2023). Signoroni et al. employed a Siamese CNN within their multi-network “DeepColony” to refine species identification on urine culture plates. Their approach first used a primary CNN to classify individual colony images with 32 considered species, before a Siamese network clusters colonies by morphological similarity. This contextual clustering recognizes when multiple colonies likely belong to the same species and adjusts predictions accordingly. DeepColony showed a top-1 identification accuracy of 90%, and the system made a final decision about which distinct species were present (Signoroni et al., 2023). This use of Siamese CNNs is particularly suited for mixed cultures. It mimics how a human notices that colonies with the same morphology on a plate are probably the same organism, thereby reducing spurious diversity in the predictions.

Another class of architectures gaining attention are object detection models and attention model. Object detectors like YOLO (You Only Look Once) and Faster R-CNN scan an entire plate image and detect each colony and identify its species microbiology (**Supplementary File 1**). This is useful for automation. Rather than cropping each colony for separate classification, the model finds colonies and labels them. Majchrowska et al. compared one-stage detectors (like YOLO) to two-stage detectors (like Faster R-CNN) and to a novel transformer-based detector (harnessing self-attention mechanisms) on the AGAR dataset. All these deep learning approaches showed strong potential to automate colony counting and classification. The novel transformer-based models, called detection transformers (DETR), have further been applied for colony analysis, too (Majchrowska et al., 2022). As datasets continue to grow, training data-intense Vision Transformer models might further improve identification by spatial relationships and long-range dependencies across an entire image simultaneously, rather than focusing on local features in isolation. **Table 1** summarizes several key studies to date, illustrating the breadth of AI approaches and their focus areas, such as bacteria vs. fungi, classification vs. detection, and variations in sample types and imaging conditions (**Table 1**). Because the included studies differ fundamentally in task definition, number and prevalence of target classes, datasets, imaging and culture conditions, reference standards, validation strategies, and reported performance measures, their numerical results are not directly exchangeable. Performance estimates should therefore be interpreted within the specific task and validation context of each study; a pooled quantitative ranking or meta-analysis was not considered methodologically appropriate. The supplementary text provides explanation how these models work in detail (**Supplementary File 1**).

**Table 1:** Selection of studies applying deep learning for microbial species identification from plate images.

Study (Year)	Summary	AI model(s) used	Validation	Sample type	Organisms	Reported limitations and future directions
(Huang and Wu, 2018),	First CNN to classify bacterial colonies by species; Demonstrated feasibility of automated colony ID.	Custom CNN (4 conv layers). Trained from scratch.	Train/test split on 300+ images from different agar types (MacConkey and Blood agar); performance measured per species. 73% overall accuracy, up to 92% for some species.	Not specified	18 bacterial species e.g., *E. coli, Staphylococcus* spp.*, Streptococcus* spp.*, Salmonella* spp.	Low precision/sensitivity for some species due to unequal class sizes, variable morphology across agar media, and limited model capacity. Performance improved with more training images. Specific future work was not explicitly reported.
(Faron et al., 2019),​	Evaluation of automated plate reader software (WASPLab) for urine cultures. Focus on growth detection and colony counting; demonstrated high sensitivity and reduced reading time.	Rule-based + image analysis (classical computer vision); preliminary ML for colony detection.	Compared to manual reading of 1000+ chromogenic urine plates (prospective).	Urine	Different bacteria. Mixed cultures on CPS chromogenic agar.	Image-only discrepancy analysis prevented confirmatory identification of detected microcolonies, and turnaround-time estimates excluded additional work-up for non–*E. coli* organisms. Future work should determine the identity and clinical relevance of microcolonies and validate specific rules for auto-reporting.
(Majchrowska et al., 2021)​	Released AGAR dataset for colony detection. Showed that deep object detectors can localize and identify colonies under varied lighting/camera conditions.	Faster R-CNN and Cascade R-CNN object detectors.	18k images of Trypticase Soy Agar. Best overall mAP: 52.3% for the higher-resolution subset and 59.4% for the lower-resolution subset; best colony-counting sMAPE: 3.81–4.86%, depending on the image subset.	Not specified	*Staphylococcus aureus*, *Bacillus subtilis*, *Pseu-* *domonas aeruginosa*, *E. coli*, and *Candida albicans.* Single and mixed cultures in dataset.	Reduced performance for overlapping, low-contrast, and densely populated colonies; no colony-level ground truth for empty or uncountable plates; and partially imbalanced acquisition subsets. Future work includes transformer-based detectors, generative models for domain generalization and additional species.
(Alouani et al., 2022),	Deep learning workflow to analyse *entire* urine culture plate images. Bypassed manual colony cropping by classifying full plate as no-growth, pure culture, or mixed, and providing presumptive ID for predominant colony type.	Two advanced deep CNN models used for analysis and interpretation of urine culture images. These CNNs were incorporated as part of the platform, BacterioSight, which performs image prepossessing and classification.	Whole-plate deep-learning classification of routine blood-agar urine cultures as negative, contaminated, or positive, using a consensus of Xception and InceptionResNetV2 models. Trained and evaluated on 114,074 images from two institutions; within-institution AUCs were ≥0.98, while cross-institution performance decreased to AUCs ≥0.90.	Urine	multiple species, but not clarified	Limitations included subjective and variable reference labels, non-identical experimental and routine review conditions, reduced cross-institution generalizability, non-standardized interpretation classes, and evaluation restricted to urine cultures. Future work included multi-dataset validation, consensus/reinforcement learning, a standardized multi-institutional gold-standard dataset, and high-confidence auto-verification with difficult-case triage.
(Signoroni et al., 2023),	“DeepColony” multi-network system for fully automated plate interpretation. Hierarchical approach. ​	Multi-stage deep learning: U-Net for colony segmentation; CNN (4-layer) for species ID; Siamese CNN for similarity clustering of colonies​; rule-based decision logic for final output.	Evaluated on >5,000 clinical urine plates in an automation setting​ (Copan WASP). High sensitivity for growth and accurate presumptive IDs; FDA-cleared for urine screening. Achieved 95.4% overall agreement with human reading of urine cultures	Urine	32 bacterial species. Urine pathogens and commensals. Multiple morphotypes per plate with mixed cultures.	Limited identification within confluent growth, residual confusion between morphologically similar or polymorphic organisms, class imbalance, and lower agreement for mixed cultures. Evaluated only for urine cultures. Future work should adapt to organism spectra encountered in other specimen types and regions.
(Pouris et al., 2025),​	*FungID* for fungal colony identification. Demonstrated that deep learning can distinguish morphologically similar fungi (e.g. *Aspergillus* spp.) by subtle color/pigment differences.	Transfer-learned CNN (VGG16) classifier; input = colony images on specialized agar.	5-fold cross-validation on 269 images of chromogenic agar. Across 10 species: Mean accuracy 89.7%, sensitivity 65.3%, precision 59.5%, F1 score 0.55, and MCC 0.54; species-specific AUC values ranged from 0.60 to 0.93.	Not specified	10 fungal species. Molds including *Aspergillus, Fusarium*, a*nd Alternaria*.	Limited pilot dataset with variable performance and confusion between morphologically similar fungal species; results were sensitive to dataset imbalance, image quality, and preprocessing, with potential overfitting and adversarial vulnerability. Future work includes larger and more diverse datasets, improved feature extraction and standardized imaging.
Weber et al. (2025) (Weber et al., 2026)	MoldVision is an AI tool for fungal colony identification. Performance comparison to human expert.	Training of three variations of VGG16 CNNs and benchmarked the best-performing model (VGG16 with dual classification heads) against LightGBM models trained on pre-extracted features and human expert assessments at various time points.	Best-performing VGG16 model achieved a mean (SD) AUROC of 92.7% +/- 1.8% and sensitivity of 68.7% +/- 2.6% across all species. Early-stage classification (1–2 days old culture) was challenging (F1-score 38.8% +/- 3.5% across all species) but improved significantly on 3–5 days old culture (F1 92.1% +/- 2.8% across all species).	Not specified	5 fungal species. *Penicillium* spp., *Aspergillus flavus* and *A. fumigatus*, *Fusarium* spp., and *Cladosporium* spp.	Limited single-centre dataset without external validation; standardized single-smartphone acquisition may restrict generalizability, and expert comparison used only static macroscopic images without microscopy. Early-growth classification was limited. Future work includes prospective multicentre validation, additional and closely related species, diverse devices and imaging conditions, newer architectures.

Major goals, methods, and findings are listed for each study in chronological order. CNN = convolutional neural network; *Siamese* CNN = twin-network for similarity learning; YOLO = You Only Look Once object detection network; ViT = Vision Transformer. Supplementary Text explains the different models **Supplementary Material**. Validation methods include hold-out test sets or cross-validation on image datasets as reported by the authors.

Combination of data-driven analysis with modern readouts. Newer technology can be combined with the task to analyse images to identify growing microorganisms. A recent study by Li et al. uses a thin-film transistor (TFT)-based image sensor coupled with deep learning algorithms. Traditional detection methods based on culturing require ≥24 hours for reliable results and include endpoint measures. This platform integrates a large field-of-view (~10 cm²) TFT image sensor, capturing time-lapse images of agar plates. The sensor collects images at 5-minute intervals, allowing for real-time monitoring. Two deep neural networks are used: the first for detecting growth and the second for classifying them as *Escherichia coli* or other coliforms. This system achieved an average CFU detection rate of 97.3% at nine hours and a classification recovery rate of 91.6% at 12 hours, saving more than 12 hours compared to conventional methods (Li et al., 2022). Other studies, such as Paquin et al., have demonstrated an average detection rate of 96.0% within 8 hours of growth using a lens free approach, in which the plate is placed directly on a CMOS sensor (Paquin et al., 2022).

Metrics suitable for image classification. To enable clinically meaningful evaluation of image-classification algorithms, performance should be reported using metrics that capture class imbalance and the real-world consequences of misclassification, not accuracy alone. **Supplementary File 2** provides an overview on the different metrics, which are commonly used in AI-based image analysis (**Supplementary File 2**). Accuracy is the proportion of correctly classified instances among all predictions and frequently reported to summarize algorithm performance in the reviewed literature. However, accuracy alone can be misleading, especially with imbalanced class distributions or when misclassifications have different clinical consequences. If one microbial species dominates a dataset, an algorithm may achieve high accuracy by predicting only the most common class, while missing rarer but clinically relevant species. This highlights the need for more nuanced metrics. Better alternatives include sensitivity (recall) and precision (positive predictive value), the F1-score, and balanced accuracy. ROC curves and AUC, particularly the area under the precision–recall curve (AUPRC), as well as confusion matrices and the Matthews correlation coefficient (MCC), provide more comprehensive assessments. In clinical contexts, metrics reflecting diagnostic utility, such as high sensitivity to minimize false negatives and high specificity to reduce unnecessary confirmatory testing, should be prioritized over accuracy alone (Amirshahi et al., 2018; Rainio et al., 2024).

## Performance and limitations of automated species identification

As shown above, the performance of CNN-based plate classification has steadily improved. Initial specificities were too low and would have resulted in many confirmatory tests. For straightforward tasks, automated species identification based on computer vision models has steadily improved in performance over time, for simple task automation, such as differentiating distinct species on selective agar, CNN models can reach overall high performance (Huang and Wu, 2018). Consequently, recent studies have moved towards more challenging application, such as species identification from multiple classes (n>20) and more detailed subclassification. Signoroni et al. achieved 91% top-1 performance on 32 species (correct if the true species corresponds to the species with the highest confidence score) and 97% top-3 performance (correct if the true species is within the *three highest* ranked species) after their similarity-based refinement. Top-1, Top-2, and Top-3 refer to *Top-n classification accuracy* based on the presumptive identification vector produced by the CNN at level 2 (Signoroni et al., 2023). Given the high implications of false negatives, sensitivity has been optimized achieving high sensitivity (>98%) and rarely miss growth. For example, plate readers in MLA systems can detect bacterial growth on urine plates with >99% sensitivity, albeit at the cost of some false positives. Specificity, corresponding to correctly identifying no-growth or insignificant growth, tends to be lower. For instance, Faron et al. reported only 68-72% specificity in distinguishing true infection from mixed/contaminant growth (Faron et al., 2019). Thus, current AI-based algorithms might over-call positives that a human would dismiss as normal flora. This is generally acceptable as the AI-based system can be used to flag plates, like a screening test, for human review while safely auto-clearing truly negative plates (Signoroni et al., 2023). Such an approach can already significantly reduce the workload within the lab. In a next step, a second layer trained for specific species identification may provide a provisional characterization (**Figure 2**). This stepwise approach could harness differently (pretrained) model architectures for these diverse tasks each emphasizing different evaluation objectives assessed by respective metrics (e.g. high sensitivity detection, but balanced classification performance as well as emphasis on uncertainty calibration of species identification to call human attention where required).

**Figure 2:**
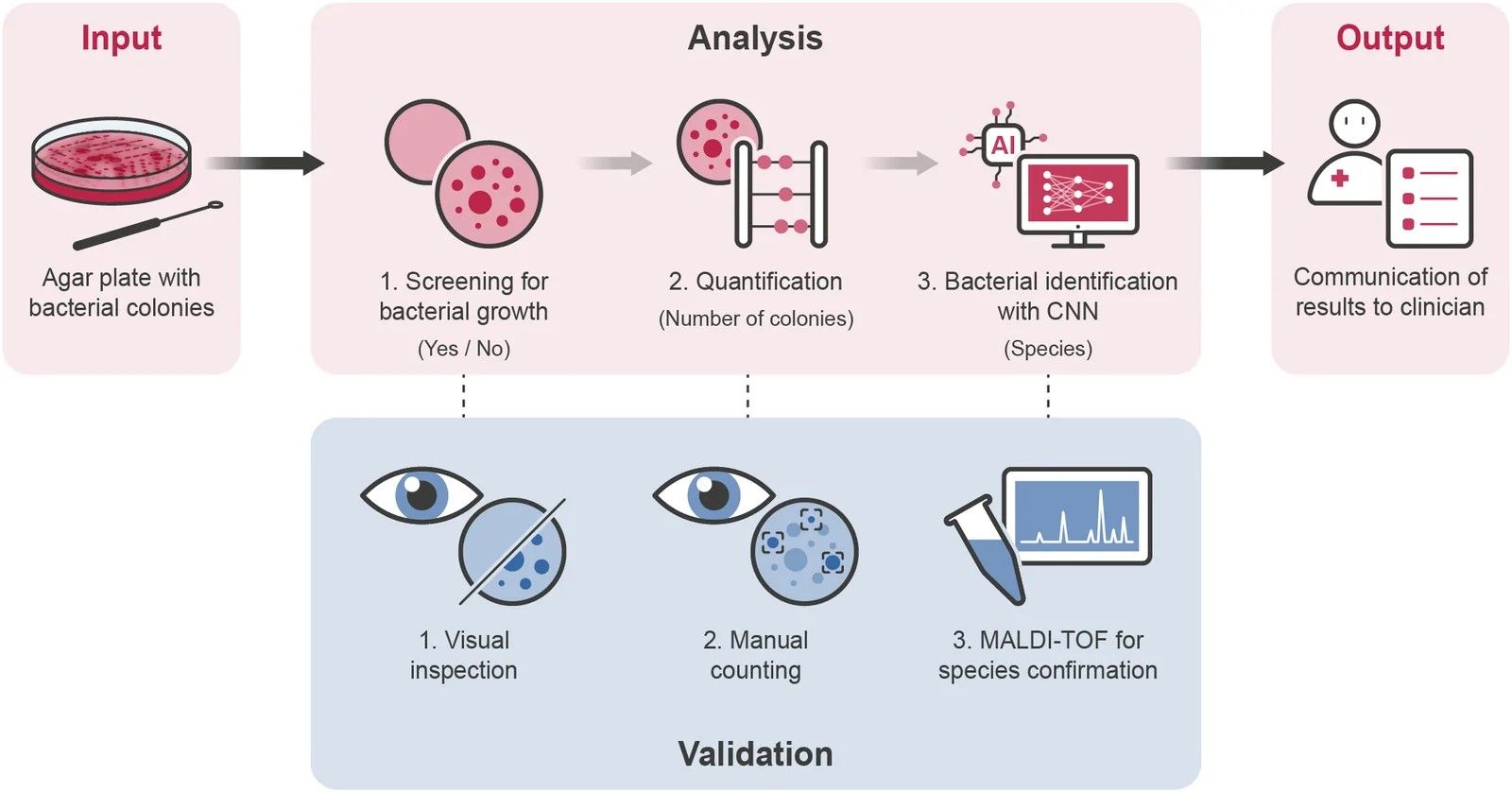
Workflow of AI-based image analysis. Left part shows the agar plate, followed by analytical steps: first, screening for bacterial growth; second, bacterial identification with a first layer of CNN. For validation, the first step is manually validated with human checking if there is growth or no growth. For the second step, MALDI-TOF MS is used to confirm the species. Third, an output is communicated via a laboratory information system to the clinician. Of note, following image acquisition, model-dependent preprocessing and colony detection or segmentation may be performed. Whole-plate classifiers can process the complete plate image directly, whereas colony-level models first localize or segment individual colonies before quantification and species classification.

A structured performance review should compare systems by task: growth detection, colony counts, and organism identification. Each step has different error modes and distinct clinical consequences. For each task, not only discrimination metrics (e.g., sensitivity/recall, specificity, precision/PPV, F1/PR-AUC) should be reported but also workflow outcomes such as reduced hands-on time, fewer unnecessary workups, faster time-to-result, and changes in confirmatory testing rates. Notably, the relative importance of these parameters varies depending on clinical and resource context. In low-resource settings, where confirmatory testing supplies may be constrained, reductions in unnecessary confirmatory testing represents the most impactful outcome. Conversely, in resource-rich environments, throughput gains may prove more valuable, alleviating pressure on laboratory personnel and accelerating patient management. Where available, we should link performance to patient impact (e.g., earlier appropriate therapy, fewer missed pathogens, improved stewardship through faster AST reads), while noting the validation context (media types, prevalence, external sites) that determines how transferable results are.

A key limitation of AI-based diagnostic systems is their potential difficulty in generalizing beyond the conditions present in their training data. For example, unusual lighting, agar types, or species not in the training set can significantly impact the performance (Signoroni et al., 2023). This sensitivity to context raises concerns about over-reliance on specific protocols or materials, and in regulated environments such as under the *in vitro* diagnostic regulation (IVDR) framework, it could lead to commercial dependencies; for instance, requiring the use of plates from a specific manufacturer. Ensuring a diverse training dataset and using techniques like data augmentation (random brightness shifts, etc.) or style transfer (Pawlowski et al., 2022) can improve generalization. Some models, e.g., the DeepColony system noted confusion between *Streptococcus mitis* vs. *S. oralis*, which are virtually identical colonies (Signoroni et al., 2023). While this may seem like a limitation, such group-level identification (e.g., viridans streptococci) is often sufficient for clinical decision-making. In ambiguous cases, rather than aiming for full automation, the AI system could be designed as a triage and decision-support tool that actively prompts follow-up testing, such as MALDI-TOF MS or even higher resolution methods. Implementing such functionality would likely require an extended system architecture, potentially combining different model types in an ensemble framework. Emerging vision–language models offer promising opportunities not only to refine predictions but also to enable interactive user engagement (Zieliński et al., 2017; Naseem et al., 2023; Okayo et al., 2025), allowing the system to explain uncertainty, suggest confirmatory diagnostics, and support microbiologists in a human-in-the-loop workflow. In this sense, AI would guide and prioritize further diagnostics rather than replace expert interpretation altogether.

Beyond the technical limitations of individual AI systems, interpretation of the current evidence base is constrained by heterogeneous reporting. Several studies lack detailed metadata on ground truth and comparator methods, often citing only “routine diagnostics” without specifying whether identification relied on biochemical tests, MALDI-TOF MS, PCR, or sequencing, nor detailing agar types or incubation conditions. As these reference standards differ in scope and accuracy, incomplete reporting hampers cross-study comparability and may bias performance estimates. Future studies should clearly document comparator methods (including discrepancy adjudication), culture conditions, and task- and organism-specific metadata to enable transparent evaluation and reproducible benchmarking.

## Integration of AI-based image analysis into automated culture systems

The uptake of AI for plate reading is closely tied to the proliferation of MLA platforms. Although systems outside of classical laboratory automation exist, such as the Azure Image capture devices and its AzureSpot Pro software (both Azure Biosystems, United States), those lack the throughput and require integration into automated incubation systems. Copan WASPLab™ (Italy) and BD Kiestra™ (Netherlands) MLA systems use robotic plate handling, automated incubators, and digital imaging stations to streamline culture processing. These platforms capture high-quality images within their workflow, creating an ideal data source for AI algorithms. Vendors have begun to incorporate AI-driven image analysis into their software suites (Faron et al., 2020; Dauwalder et al., 2021; Uwamino et al., 2022). For example, Copan’s PhenoMATRIX™ AI and BD’s Kastle™ (Urine Culture App) apply AI to identify plates with no significant growth, count colony forming units (CFUs), and recognize colony colour types. Clever Culture Systems’ APAS Independence (Automated Plate Assessment System) was the first to earn the United States Food and Drug Administration (FDA) clearance for an AI-based plate reader in 2019, focused on urine cultures (Glasson et al., 2017). APAS uses a classifier to categorize urine plate images as “no growth,” “significant growth,” or “mixed/contaminated” according to predetermined rules based on a decision tree. Studies showed APAS and similar tools can reduce the workload on microbiologists: one report found an approximately 80% reduction in hands-on review for negative urine cultures by automation plus AI screening (Glasson et al., 2017). Users of automated systems reported faster time-to-result and reduced workload. In one lab, incubation-to-report time was shortened for urine cultures on average by four hours (Faron et al., 2019), largely by earlier flagging of positive plates.

While current commercial systems stop short of fully automated species identification, focusing mainly on flagging plates and enumerating colonies, they lay the groundwork for it. The DeepColony research foreshadows the next generation of PhenoMATRIX™ AI that can propose species identification (Signoroni et al., 2023). In routine use, the commercially available AI systems (Copan WASPLab™ and BD Kiestra™) can highlight pink colonies on chromogenic plates used in urine culture and label it as “*E. coli*” with high confidence when chromogenic agar has been validated accordingly. Another immediate use is recognizing *mixed cultures* vs. pure (Signoroni et al., 2023). Current systems often operate with a combination of learned models and rule-based expert logic. For example, an AI might classify colonies into morphotypes, then apply if-then rules encoded by clinical experts e.g. “if >100 CFU of a single morphotype, report as pure culture; if two morphotypes present, flag as mixed”. This hybrid approach is seen in PhenoMATRIX™ AI and Kiestra’s intelligent software (Dauwalder et al., 2021).

Implementation challenges. Current MLA platforms and AI-enabled plate-reading solutions are not yet suitable for low resource settings deployment in automation mode because they require high upfront investment, reliable service contracts, stable electricity and IT infrastructure, and ongoing access to proprietary consumables and spare parts (Lippi and Da Rin, 2019). Deployment also depends on specialized installation, calibration, and trained staff for maintenance, quality management, and regulatory compliance resources often scarce outside centralized reference laboratories. Vendor lock-in, across hardware–software–media combinations, further creates logistical vulnerabilities when supply chains are disrupted. In contrast, smartphone-based imaging paired with lightweight, open-source algorithms could offer a more affordable, scalable, and locally maintainable pathway for AI-assisted culture interpretation in resource-limited settings (Koydemir and Ozcan, 2017). However, even smartphone approaches still require rigorous quality control, external validation, and fit-for-purpose regulatory standards to ensure precision and patient safety.

In high-resource settings, MLA and AI-driven image recognition are strongly promoted because they help laboratories manage rising sample volumes, cost pressure, and shortages of skilled technologists by standardizing repetitive tasks (e.g., plate transport, imaging, triage of “no-growth” cultures) and improving turnaround times. Successful implementation, however, requires proactive change management: staff should be involved early, trained, and supported so automation is seen as augmentation rather than replacement (Cherkaoui et al., 2020; Trigueiro et al., 2024). Without transparent communication and clear role definitions, employees may fear job loss to “AI systems” undermining adoption and quality. Building AI-related expertise quickly can also be challenging for lab technicians (Tong et al., 2025). Embedding technologists and microbiologists in validation, rule-setting, exception handling, and continuous quality monitoring preserves expertise, maintains trust, and helps ensure safe, sustainable implementation.

Regulatory aspects. A common question in the adoption of AI-based image recognition in microbiology is how well these systems agree with trained human experts? In the validation of DeepColony’s on 2,623 urine cultures, the AI demonstrated an overall agreement of 95.4% with manual interpretations, including near-perfect concordance (99.2%) in identifying truly no-growth plates. The biggest discrepancy occurred in defining “mixed growth”. The AI sometimes “overcalled” clinically insignificant mixtures as significant, leading to 77% agreement on the subset of plates deemed contaminated by human microbiologists (Signoroni et al., 2023). This is a conservative error (falsely elevating some reports), which is preferable to missing a pathogen.

AI-based image analysis must undergo rigorous IVDR/FDA validation before routine use. Human colony identification on selective chromogenic agar without confirmatory testing is already approved in some cases (e.g., E. coli on chromogenic “orientation” agar from BD, bioMérieux, or Oxoid). Both systems, Copan and BD Kiestra, allow for identification of E. coli on validated chromogenic plates in urinary culture. PhenoMATRIX is IVDR conform registered as a class C product under IVDR. Pairing automated readers with IVDR-approved chromogenic plates could therefore be an early route to species identification. Under IVDR, pre-trained “locked” models are more realistic, as ongoing re-training/self-learning is restricted. Any software update or new version will likely require renewed validation, supported by standardized in silico control sets (e.g., curated images of closely related bacterial and fungal colonies across multiple agar media) used as internal benchmarks. Such quality controls help ensure updates do not degrade performance.

## Limitations of this review

This review has several limitations. First, although we aimed to provide a comprehensive overview of AI-based species identification from agar plate images, the field is rapidly evolving, and newly published preprints or commercial developments may not have been captured at the time of writing. Second, the reviewed studies are highly heterogeneous with respect to datasets, imaging setups, agar types, organism panels, and performance metrics, which limits direct comparability and precludes quantitative synthesis or meta-analysis. Many studies were conducted in single-center settings with limited external validation, often using curated datasets that may not reflect real-world variability in lighting, incubation time, mixed cultures, or rare pathogens. In addition, reporting standards varied considerably, particularly regarding ground-truth definition, discrepancy adjudication, and detailed metadata on culture conditions. This heterogeneity may introduce publication and performance bias, as studies reporting strong results under controlled conditions are more likely to be published. Finally, the rapid integration of AI tools into commercial laboratory automation platforms means that some proprietary systems cannot be evaluated in full technical transparency, limiting independent benchmarking. Future reviews would benefit from standardized reporting frameworks, harmonized benchmarking datasets, and multi-center validation studies to enable more robust comparison across approaches.

## Future perspectives and research priorities

While high-end automation is transforming centralized microbiology laboratories, a parallel movement towards smartphone-based and other low-cost imaging approaches is gaining traction (Koydemir and Ozcan, 2017; Ong and Poljak, 2020; Pascucci et al., 2021). However, an important gap remains between promising performance in controlled proof-of-concept studies and safe, generalisable implementation in routine diagnostic microbiology. Future research should therefore address three central gaps: external generalisability, reliable interpretation of clinically complex cultures, and the lack of standardised datasets and evaluation frameworks.

Research gap 1: External generalisability and prospective clinical validation. Most published models have been developed retrospectively in single centres using a limited combination of agar media, incubation conditions, imaging devices, and local organism distributions. Performance may decrease when models are transferred to other laboratories. For example, BacterioSight achieved AUC values of ≥0.98 when trained and tested within the same institution, but cross-institution performance decreased to ≥0.90 (Alouani et al., 2022). Future studies should therefore use prospective, multicentre designs and deliberately assess variation in cameras, lighting, agar media and manufactures, incubation times, specimen types, and regional organism prevalence. Training and validation sets should be separated to avoid data leakage, and validation cohorts should include rare organisms, unusual phenotypes, mixed cultures, and technically suboptimal images. In addition, studies should report calibration, confidence intervals, subgroup performance, failure, and changes in performance over time.

Research gap 2: Reliable interpretation of clinically complex cultures. Many current models have been trained predominantly on isolated colonies, pure cultures, or well-defined screening tasks. Their performance is less established for confluent growth, polymicrobial specimens, morphologically similar organisms, contamination, and colonies at early stages of development. DeepColony, for example, could not reliably identify species within confluent areas and showed lower agreement with technologists for mixed or contaminated cultures (Signoroni et al., 2023). Colony maturity is also important: in MoldVision, the overall F1 score increased from 38.8% during days 1–2 to 92.1% during days 3–5, demonstrating that biologically immature phenotypes remain difficult to classify even with advanced models (Weber et al., 2026). Future systems should therefore move beyond isolated classification tasks towards hierarchical workflows combining growth detection, colony localisation or segmentation, enumeration, and contextual species identification. Models should quantify uncertainty and be able to abstain from interpretation when confidence is insufficient, referring ambiguous cultures for expert review or confirmatory testing. Evaluations should assess not only analytical accuracy but also clinically relevant outcomes such as time to result, frequency of confirmatory testing, unnecessary work-up, and effects on antimicrobial decision-making.

Research gap 3: Standardised datasets, ground truth, and performance reporting. Publicly accessible datasets of macroscopic microbial culture images remain scarce and generally cover only a limited number of organisms and acquisition conditions. The AGAR dataset represents an important resource, with 18,000 plate images and more than 336,000 annotated colonies, but it includes only five microorganisms under defined experimental conditions (Majchrowska et al., 2021). Most clinical datasets remain local or proprietary, and studies differ substantially in their unit of analysis, annotation granularity, class distribution, culture media, incubation conditions, reference identification methods, and treatment of mixed growth. Ground truth is also inconsistently defined and may be based on routine technologist interpretation, expert consensus, MALDI-TOF MS, or molecular diagnostics. Future collaborative datasets should therefore include multiple laboratories, geographical regions, imaging platforms, agar types, growth stages, pure and polymicrobial cultures, confluent growth, artefacts, and negative plates. Images should be linked to traceable metadata and an appropriate reference identification. Common benchmark partitions and task-specific reporting standards are required for growth detection, colony enumeration, object detection, species identification, and whole-plate interpretation. Class-specific sensitivity, precision, F1 score, calibration, confidence intervals, and failure rates should be reported alongside overall metrics.

Smartphone based applications. Against this background, smartphone-based microbiology holds promise for smaller laboratories and resource-limited settings. Modern smartphones provide high-resolution cameras, substantial onboard computing power, and increasingly efficient AI accelerators, permitting standardised plate imaging and local execution of lightweight CNNs. In regions without access to MALDI-TOF MS or full laboratory automation systems, such approaches could support growth detection, colony enumeration, and presumptive identification at comparatively low cost. Early microbiology applications are already available (Minoi et al., 2016; Austerjost et al., 2017; Moucka et al., 2022; Taithong et al., 2022). Moucka et al., for example, compared smartphone applications for bacterial colony counting and identified substantial differences in performance between applications (Moucka et al., 2022), underlining the need for standardised acquisition and independent validation. Extending smartphone-based colony enumeration to presumptive species identification is a logical next step. An application could guide users through image acquisition using a defined background, illumination, camera angle, and plate-to-camera distance, and then analyse the images locally or through a cloud-hosted models. Outputs could include ranked identification probabilities, uncertainty estimates, and alerts for mixed or atypical growth, enabling more targeted confirmatory testing. Frameworks such as TensorFlow Lite allow lightweight models to operate directly on smartphones without continuous internet access (Gorospe et al., 2021). Selective and chromogenic media may be particularly suitable for initial applications because organisms can produce distinctive colour and growth patterns, such as lactose-fermenting pink *Escherichia coli* on MacConkey agar, yellow *Vibrio cholerae* on TCBS agar, green *Candida albicans* on appropriate chromogenic media. Interactive AI systems could additionally request further images, different viewing angles, incubation information, or colony characteristics when the initial prediction is uncertain. Nevertheless, smartphone systems require the same external validation, quality control, and clearly defined intended use as high-end laboratory automation.

Alternative optical readouts may further improve discrimination beyond conventional red–green–blue imaging. Elastic light scattering has shown considerable potential for label-free colony identification. Bae et al. reported average classification accuracies of 95–100% using a compact laser- and Charge-coupled device (CCD)-based system (Bae et al., 2012), while Genuer et al. achieved a correct identification rate of 94% after incorporating backscatter detection (Genuer et al., 2016). Schultz et al. demonstrated a best correct identification rate of 94% for distinguishing *Staphylococcus aureus* from non-*S. aureus* isolates after six hours of incubation (Schultz et al., 2018). More recently, Doh et al. introduced a smartphone-based reflective elastic light-scattering system that captures laser-illuminated colony backscatter patterns and achieved classification accuracies above 94% for several clinically relevant bacteria (Doh et al., 2022).

Hyperspectral and lens-free imaging offer additional avenues. Yoon et al. combined visible near-infrared hyperspectral imaging with machine learning to classify Shiga toxin-producing *E. coli* serotypes and achieved a mean classification accuracy of 95.06% (Yoon et al., 2013). Douarre et al. used lens-free imaging, in which the culture plate is placed directly on the imaging sensor, and reported a colony identification rate of 91.7% (Douarre et al., 2024). Galudec et al. combined lensless and infrared imaging to classify ten bacterial strains with an accuracy of 94.4% ± 1.6% (Le Galudec et al., 2025). These technologies may capture biochemical or structural information that is not visible in standard colour images. Their added value should, however, be assessed in prospective head-to-head studies using identical specimens, reference methods, and clinically meaningful endpoints rather than through internal classification accuracy alone.

Implementation strategies. Regardless of the imaging modality, AI-based plate reading should initially be implemented as a risk-stratified decision-support system with a clearly defined intended use, such as no-growth triage, contamination flagging, colony enumeration, or presumptive identification. Clinical implementation requires standardised imaging protocols, integration with laboratory information systems and standard operating procedures, traceable ground truth, predefined decision thresholds, explicit rules for mixed and atypical growth, staff training, and continuous quality monitoring. Regulatory implementation will require robust multicentre validation and strict change control for software updates, favouring controlled or “locked” models whose performance can be verified against standardised image panels. Priority areas therefore include harmonised benchmarking datasets, task-specific reporting, uncertainty calibration, drift surveillance, and multimodal or interactive systems that combine image analysis with laboratory metadata and expert interpretation. Smartphone-based tools may substantially broaden access, but they must meet the same validation and quality-assurance standards as systems deployed within full laboratory automation.

The key future objective is therefore not simply to obtain higher accuracy under controlled conditions, but to demonstrate reliable, auditable, and clinically useful performance across laboratories, acquisition systems, organism spectra, and complex real-world cultures.

## Conclusion

AI-based image analysis, particularly from the realm of computer vision architectures, significantly enhances microbial species identification by detecting subtle visual features often missed by human observers. While deep learning models already approach expert-level performance, further validation, standardization of metadata reporting, and regulatory approvals are needed before widespread clinical implementation. Besides MLA systems, smartphone-based image acquisition may be an option from low resource settings, but also here in-depth validation is required. Nonetheless, AI promises substantial benefits, accelerating diagnostics in high-resource labs and expanding microbiological capabilities in resource-limited settings.
