# Supplementary material for: State-of-the-art in automated image-based species identification systems
Source: Front Cell Infect Microbiol. 2026 Oct 20;16:1818023. doi: 10.3389/fcimb.2026.1818023 (PMC13728494; doi:10.3389/fcimb.2026.1818023)
Supplement: Supplementary file 1 [file SupplementaryFile1.docx]

**Supplementary Text 1. Deep Learning Techniques for Microbial Image Analysis**

This supplementary text provides a practical primer on the main deep learning techniques referenced in this review for **image-based analysis in medical microbiology**. For each model family (classification, segmentation, object detection, transformer-based vision, lightweight edge models, and multimodal large language models), we briefly summarize the core operating principle and explain how it maps to **colony-based species identification**. In some examples, we will use other typical lab examples such as **microscopy-supported recognition** and **image-derived antimicrobial susceptibility readouts** (e.g., zone or growth-pattern analysis). We also highlight common hybrid and robustness strategies, such as **rule-guided post-processing**, **transfer learning**, and **data augmentation/style transfer**.

**Convolutional Neural Networks (CNNs)**

CNNs learn directly from images by sliding small filters across pixels to detect increasingly complex visual patterns (edges → textures → shapes). CNNs are typically trained on cropped colony patches or plate regions to classify broad organism groups or specific species based on colony morphology, color, and texture. This is well-suited to agar-plate identification because colonies are visual “objects” with reproducible phenotypes under standardized illumination. A practical example is a culture-plate CNN pipeline that compared multiple CNN backbones for classifying colonies into clinically relevant categories [1].

*Residual Networks (ResNet).* ResNets are CNNs with skip connections (“residual blocks”) that let gradients flow through very deep networks, improving training stability and accuracy. In plate-image workflows, ResNet backbones are often used either for direct colony classification or as a strong feature extractor after colonies/regions have been segmented. This is helpful when subtle morphological differences matter (e.g., early vs. late colony appearance, or look-alike taxa) because deeper models can learn more discriminative feature hierarchies. A representative microbiology use case is a pipeline where ResNet50 was applied downstream of colony segmentation to automatically count colonies and handle adherent/clustered growth [2].

*VGG architectures (VGG16)*. VGG-style CNNs use a simple, uniform design: many stacked 3×3 convolutions that progressively build rich visual features while keeping the architecture easy to implement and benchmark. VGG16 is commonly used as a robust baseline for colony-image identification, especially when paired with standardized imaging (top/bottom views, time series). This architecture can learn discriminative morphology and color cues that expert readers also rely on (e.g., sporulation, texture, colony margins). A direct example is smartphone-based mold identification using VGG16 variants trained on sequential culture-plate images across days of growth [3].

*AlexNet* (incl. AlexNet/ResNet ensembles). AlexNet is an earlier CNN architecture that introduced large-scale deep learning for images using stacked convolutions and fully connected layers; today it is often used as a historical baseline. In colony-image tasks, AlexNet may underperform newer backbones but remains useful to show the value of modern architectures and training practices. “Ensembles” combine predictions from multiple CNN backbones (e.g., AlexNet + ResNet-like models) to reduce variance and improve robustness across changing lighting, agar types, and camera setups, common failure modes in plate imaging. In microbiology-image classification, ensemble approaches have been explored to improve stability and accuracy when single models are sensitive to acquisition variability ([1, 4]).

*Siamese CNN / Siamese networks* (similarity learning / contextual grouping). Siamese networks process two images through identical CNN “twin” branches to produce embeddings, then learn a distance metric so that same-class images cluster together while different classes separate. In agar-plate interpretation, this is powerful for *contextual grouping*: colonies that look alike on the same plate can be clustered, and identification can be refined by enforcing intra-plate consistency. This mirrors the human workflow of grouping morphologically similar colonies before deciding what is clinically relevant. A clinical example is DeepColony, which explicitly uses a Siamese CNN stage to update colony IDs using similarity-driven contextual grouping [5].

*U-Net* (colony / growth-zone segmentation). U-Net is an encoder–decoder (called “autoencoder” in the literature) CNN designed for pixel-wise segmentation, using skip connections to combine fine detail (edges) with deep semantic features. U-Net-like models are used to segment colonies, plate regions, or growth/inhibition areas, turning images into measurable masks before classification or rule-based interpretation. This is particularly relevant for resistance-related imaging where the key signal is spatial (e.g., growth vs no growth, inhibition zones, interaction gradients). A concrete example is automated segmentation of bacterial growth on CombiANT assay plates to quantify antibiotic interactions with high precision from smartphone photos [6].

*Faster R-CNN*. Faster R-CNN is a two-stage detector: it first proposes candidate regions (Region Proposal Network), then refines classification and bounding boxes for higher accuracy. In microbiology images, this is attractive when precise localization matters, e.g., accurately delineating inhibition zones and identifying antibiotic disks so zone diameters can be computed reliably. This directly supports resistance analysis from plate images by reducing reader variability and enabling standardized measurements. One application trained a Faster R-CNN (ResNet-50 backbone) to detect plates, disks, and inhibition zones from Kirby–Bauer images in an AI-driven “Disc Diffusion Reader” [7].

*Cascade R-CNN*. Cascade R-CNN extends Faster R-CNN by chaining multiple detection heads with increasing intersection over union (IoU) thresholds, progressively improving localization quality and reducing false positives. For agar-plate microbiology, this helps with crowded scenes: overlapping colonies, variable illumination, and small objects near plate edges. Better localization translates into better colony counting and cleaner inputs for downstream identification models, especially when species labels depend on correctly separated objects. A microbiology example used Cascade R-CNN (alongside Faster R-CNN) trained on synthetic + real Petri-dish images to improve colony detection performance [8].

*Lightweight CNN variants* (e.g., MobileNet, ShuffleNet). Lightweight CNNs reduce parameters and floating point operations per second (FLOPs) using architectural tricks (bottlenecks, grouped convolutions, compact blocks) to run on edge devices or high-throughput automation lines. They are useful for on-bench or smartphone-based microbiology image analysis, where compute and memory are constrained. They often serve as fast classifiers after detection/segmentation, such as recognizing antibiotic disk labels or coarse colony categories. Examples include MobileNet/ShuffleNet performing strongly in colony classification benchmarks and MobileNetV2 being used for disk classification in automated antibiotic susceptibility testing image analysis [1, 7]. As an example, *Depthwise separable CNNs* is the core idea behind MobileNet-family models. Depthwise separable convolutions split standard convolution into (i) a depthwise spatial convolution per channel and (ii) a pointwise (1×1) mixing step, dramatically reducing computation.

**Object detection networks (general concept)**

Object detection models jointly localize targets (bounding boxes) and classify them, enabling “whole-plate” interpretation rather than single-crop classification. In medical microbiology imaging, detectors can find colonies, antibiotic disks, inhibition zones, or smear objects, enabling enumeration, targeted picking, and automated measurement. This matters clinically because plates images often contain many relevant objects whose spatial relationships carry meaning (mixed flora, confluent growth, zone edges). Disk-diffusion pipelines provide clear examples where detectors identify plates/zones/disks to automate downstream susceptibility interpretation [7, 8].

*You Only Look Once (YOLO).* YOLO is a one-stage detector that predicts bounding boxes and classes in a single pass, making it fast and suitable for real-time or high-throughput plate reading. In agar imaging, YOLO-style models are used to detect/count many small colonies efficiently, including early micro-colonies that are easy to miss manually. This supports both identification workflows (finding candidate colonies for classification) and quantification workflows (CFU counting, screening growth). A recent example is YOLOv8-nano which improve small colony detection while reducing computational cost, supporting deployment in resource-constrained settings [9].

**Transformer-based vision models (general)**

Vision transformers (ViT) complement convolutions with self-attention, allowing models to capture long-range dependencies across the entire image. In microbiology imaging, this is especially useful for large-field views like whole-slide microscopy, where sparse organisms and global context (background, stain variability, scanning artifacts) influence interpretation. These models can reduce reliance on manual patch annotations by learning representations over large images at scale. A representative example is a transformer-based model for Gram-stained whole-slide classification into clinically relevant morphology categories (e.g., Gram-negative rods vs Gram-positive cocci) [10].

*Vision Transformers*. A ViT splits an image into fixed-size patches, embeds them like “tokens” using a convolutional layer and then uses multi-head self-attention to learn relationships among patches. In medical microbiology microscopy, this can improve recognition of distributed patterns (e.g., parasites across a smear) and reduce sensitivity to local noise by leveraging global context. ViTs are particularly appealing when morphology varies subtly with lifecycle stage or staining differences, because attention can highlight discriminative regions dynamically. An example applied a ViT framework to thin blood smear images for *Plasmodium vivax* lifecycle-stage classification [11].

*Transformer-based object detectors using self-attention* (e.g., DETR / RT-DETR). DETR-style detectors treat object detection as set prediction, using attention to match predicted objects to ground truth without hand-crafted anchor design. In microbiology imaging, this can help in dense or noisy fields (e.g., blood smears), where classical heuristics and post-processing may fail. Real-time variants (RT-DETR) can be deployed on low-cost devices, which is relevant for microscopy-based infectious disease diagnostics in low-resource settings. A proof-of-concept used RT-DETR on thin blood smears to discriminate *Plasmodium* species at the patient level [12].

*Multimodal large language models (LLMs)*. Multimodal LLMs accept images plus text instructions and generate structured interpretations, which can be used to turn image-derived measurements into microbiology-relevant conclusions. This is most compelling when paired with strict guidelines: the model can “explain” and apply interpretive rules rather than only classify pixels. A published example validated a customized generative pre-trained transformer (GPT)-agent for pre-classification of disk diffusion results to flag likely ESBL/AmpC/carbapenemase phenotypes, highlighting both promise and specificity limitations. This illustrates why these systems should be positioned as decision-support with careful validation and oversight, not autonomous interpretation [13].

**Hybrid approaches: rule-based / expert-rule logic combined with ML outputs**

Hybrid pipelines combine machine learning (detection/segmentation/classification) with explicit microbiology rules (breakpoints, quality control constraints, “if–then” expert logic) to produce auditable outputs. In plate imaging, rules can enforce domain constraints (e.g., plausible zone diameter ranges, expected disk layouts, or EUCAST/CLSI interpretation steps) and reduce clinically unsafe errors from raw model predictions. This is especially relevant for resistance analysis, where the last step is not “a mask” but a categorical interpretation (categories such as susceptiple, intermediate or resistant, or mechanism flags) that must align with standards. The EUCAST-GPT-expert work explicitly integrates EUCAST guidance and breakpoint tables into an agentic system to interpret disk diffusion in a guideline-constrained way [13].

**Transfer learning**

Transfer learning starts with a network pretrained on a large dataset and then fine-tunes it on a smaller, task-specific microbiology dataset. For example, using all backbones stated before (ResNet, AlexNet, MobileNet) then fine-tune them to microbiology is a transfer learning pipeline. This is highly practical for clinical microbiology imaging, where labeled colony or microscopy datasets are often limited and expensive to curate, yet pretraining provides strong generic visual features. In our context, transfer learning supports rapid development of reliable classifiers for fungi or bacteria with fewer labeled examples, while maintaining performance across imaging variability. A clinical example used transfer learning plus attention mechanisms to classify filamentous fungi from routine microscopic preparations in the clinical lab [14].

**Data augmentation and style transfer** (robustness/generalization)

Data augmentation deliberately perturbs training images (rotation, blur, brightness, color shifts) to make models robust to real-world variation in cameras, lighting, agar types, and incubation effects. Style transfer goes further by generating synthetic images that preserve labels/geometry but change “appearance style” (e.g., illumination/texture), expanding datasets without manual annotation. This is especially usefull in microbiology where models trained in one lab often fail elsewhere unless domain shift is addressed. A microbiology-specific example generated annotated synthetic Petri-dish images using classic computer vision plus neural style transfer to train object detectors for localizing/segmenting/classifying microbial colonies [8].

**Reference**

1.	Kong, F., et al., *Implementation of convolutional neural networks for microbial colony recognition.* Microbiol Spectr, 2025. **13**(9): p. e0288524.

2.	Cao, L., et al., *U(2)-Net and ResNet50-Based Automatic Pipeline for Bacterial Colony Counting.* Microorganisms, 2024. **12**(1).

3.	Weber, L., et al., *Advancing mold identification in the routine laboratory, performance of smartphone-based imaging and a newly developed convolutional neural network.* Microbiol Spectr, 2025: p. e0292425.

4.	Ahmad, F., et al., *Deep ensemble approach for pathogen classification in large-scale images using patch-based training and hyper-parameter optimization.* BMC Bioinformatics, 2023. **24**(1): p. 273.

5.	Signoroni, A., et al., *Hierarchical AI enables global interpretation of culture plates in the era of digital microbiology.* Nat Commun, 2023. **14**(1): p. 6874.

6.	Hallstrom, E., et al., *CombiANT reader: Deep learning-based automatic image processing tool to robustly quantify antibiotic interactions.* PLOS Digit Health, 2025. **4**(7): p. e0000669.

7.	Nguyen, H.B., et al., *Disc Diffusion Reader: an AI-powered potential solution to combat antibiotic resistance in developing countries.* J Infect Dev Ctries, 2025. **19**(5): p. 699-711.

8.	Pawlowski, J., S. Majchrowska, and T. Golan, *Generation of microbial colonies dataset with deep learning style transfer.* Sci Rep, 2022. **12**(1): p. 5212.

9.	Wang, M., et al., *Colony-YOLO: A Lightweight Micro-Colony Detection Network Based on Improved YOLOv8n.* Microorganisms, 2025. **13**(7).

10.	McMahon, J., et al., *A novel framework for the automated characterization of Gram-stained blood culture slides using a large-scale vision transformer.* J Clin Microbiol, 2025. **63**(3): p. e0151424.

11.	Sengar, N., R. Burget, and M.K. Dutta, *A vision transformer based approach for analysis of plasmodium vivax life cycle for malaria prediction using thin blood smear microscopic images.* Comput Methods Programs Biomed, 2022. **224**: p. 106996.

12.	Guemas, E., et al., *Automatic patient-level recognition of four Plasmodium species on thin blood smear by a real-time detection transformer (RT-DETR) object detection algorithm: a proof-of-concept and evaluation.* Microbiol Spectr, 2024. **12**(2): p. e0144023.

13.	Giske, C.G., et al., *GPT-4-based AI agents-the new expert system for detection of antimicrobial resistance mechanisms?* J Clin Microbiol, 2024. **62**(11): p. e0068924.

14.	Huang, T.S., et al., *Attention-Guided Transfer Learning for Identification of Filamentous Fungi Encountered in the Clinical Laboratory.* Microbiol Spectr, 2023. **11**(3): p. e0461122.
