# Supplementary material for: State-of-the-art in automated image-based species identification systems
Source: Front Cell Infect Microbiol. 2026 Oct 20;16:1818023. doi: 10.3389/fcimb.2026.1818023 (PMC13728494; doi:10.3389/fcimb.2026.1818023)
Supplement: Supplementary file 2 [file SupplementaryFile2.docx]

**Supplementary Text 2: Evaluation metrics in image-based microbial identification**

Reliable evaluation of image-based microbial identification models requires more than a single summary statistic, because microbiology datasets are often imbalanced, clinically heterogeneous, and may include polymicrobial growth. As a result, different metrics capture different, and sometimes complementary, aspects of model behavior. This includes as examples the ability to detect rare but important species, avoid false positives, or maintain performance across decision thresholds. This supplementary text provides a microbiology-oriented explanation of commonly reported classification metrics and how to interpret them. The goal is to support transparent reporting and facilitate meaningful comparison across studies and deployment settings. A general overview on metrics used in machine learning was recently published [1].

In image-based microbial identification using deep learning, model performance is quantified by a range of evaluation metrics. Key metrics include *Accuracy*, *Balanced Accuracy*, *Precision* (Positive Predictive Value), *Sensitivity* (Recall), *F1-Score*, *AUROC* (Area Under the Receiver Operating Characteristic Curve), *AUPRC* (Area Under the Precision-Recall Curve), the *Confusion Matrix*, and the *Matthews Correlation Coefficient* (MCC). Each of these metrics offers unique insights into classification performance, especially under conditions common in microbiological data (e.g. class imbalance or polymicrobial samples). Below, we define each metric and discuss their interpretation in the context of microbial image classification (covering both bacterial and fungal examples).

***Accuracy*.** Accuracy measures the overall proportion of correct predictions among all samples **[2, 3]**. In a binary classification, it is defined as $\left( TP+TN \right)/\left( TP+TN+FP+FN \right)$, where *TP* is true positives, *TN* true negatives, *FP* false positives, and *FN* false negatives. For multi-class problems, accuracy is the number of correctly classified images divided by the total number of images. While accuracy is intuitive and widely used, it can be misleading in imbalanced datasets [4]. For example, if images of *Streptococcus pneumoniae* vastly outnumber other classes, a model predicting everything as *S. pneumoniae* might yield high accuracy but would fail to detect other species. Indeed, accuracy may remain high by favoring the majority class, ignoring minority classes [5, 6]. In such cases, complementary metrics are needed to assess performance on under-represented microbes.

***Balanced Accuracy*.** Balanced accuracy is designed to handle class imbalance by averaging the accuracy on each class. In binary terms, it is the arithmetic mean of sensitivity and specificity [10, 11]. Equivalently, it can be seen as averaging the recall for the positive class and the recall for the negative class. For multi-class problems, balanced accuracy often refers to the average of per-class recall (mean sensitivity across classes). By weighting each class equally, balanced accuracy ensures performance on minority classes contributes fairly to the overall score [12]. For example, if a deep learning model classifies ten bacterial species where one species is rare, a high balanced accuracy means the model is performing well on all species, not just the common ones. A balanced accuracy of 80% implies that, on average, the model is 80% sensitive on each class (which also implicitly accounts for a high true-negative rate on each class). This metric is more robust to class imbalance than raw accuracy; a trivial classifier that predicts only the majority class would have high regular accuracy but low balanced accuracy (since recall on minority classes would be 0%). In microbiological datasets where some pathogens (e.g., *E. coli*) are far more frequent than others, balanced accuracy is a more trustworthy indicator of overall model performance.

***Precision* *(Positive Predictive Value).*** Precision is the fraction of positive predictions that are truly correct [7]. Formally, precision = $TP/\left( TP+FP \right)$. It answers the question: “Of all images the model *calls* a given organism, how many actually *are* that organism?”. High precision means that when the model predicts a microbe (e.g., calling a colony *Aspergillus*), it is usually correct. In microbiology, precision is crucial when false positives are costly – for instance, misidentifying a non-pathogenic colony as a pathogen could lead to unnecessary treatments. A model with precision = 0.90 for *S. pneumoniae* means 90% of the colonies predicted as *S. pneumoniae* were truly that species (10% were false alarms). Precision tends to be lower if the model frequently confuses similar-looking species, increasing FP counts.

***Sensitivity (Recall)*.** Sensitivity (also called recall or true positive rate) is the fraction of actual positive instances that the model correctly identifies [3, 8]. It is defined as $TP/\left( TP+FN \right)$. In other words, recall answers: “Of all the images that truly contain a given microbe, how many did the model detect?”. High recall indicates the model misses few positives. For example, a recall of 95% for *Aspergillus* means the model detects 95 out of 100 actual *Aspergillus* cases, missing only 5. In microbiological terms, sensitivity is critical for not overlooking pathogens: a low recall for *S. pneumoniae* would mean many pneumococcal colonies go undetected (high FN). There is often a trade-off between precision and recall: a very sensitive model may trigger more false positives, lowering precision, whereas a very precise model might be conservative and miss some positives (lower recall).

***F1-Score.*** The F1-score is the harmonic mean of precision and recall [9]. It provides a single metric that balances both, calculated as $2\times\frac{\text{Precision}\times\text{Recall}}{\text{Precision}+\text{Recall}}$. An F1-score is high only when both precision and recall are high; it punishes extreme imbalances between them. This is useful in microbiology when we desire a balance. For instance, we want to both catch as many true pathogens as possible (high recall) and avoid the same time false alarms (high precision). F1 is particularly informative for imbalanced datasets or when positive cases are rare, as it will reflect poor performance even if accuracy is high. For example, if a model identifying *Candida* in blood culture images has precision 80% and recall 80%, its F1 ≈ 0.80. But if precision is 95% and recall 50%, F1 will be much lower (≈0.66), highlighting the drop in recall despite high precision.

***AUROC (Area Under the Curve of Receiver Operative Characteristics).*** The ROC curve plots the True Positive Rate (sensitivity) against the False Positive Rate (1 − specificity) at various classification thresholds. The AUROC is the area under this curve, summarizing the model’s ability to distinguish between classes across all threshold settings [3]. AUROC is usually also called AUC-ROC or simply AUC, they are used as synonyms in the literature. An AUC of 1.0 indicates perfect separation of classes, whereas 0.5 indicates no better than random, basically flipping a coin. In microbial identification, AUROC is often used for binary classification tasks (e.g., pathogen vs. non-pathogen, or detecting presence vs. absence of a species). A high AUROC means that the model can achieve a good trade-off between sensitivity and specificity. As an example, one could set a threshold to attain both high sensitivity and specificity simultaneously. However, in highly imbalanced scenarios, AUROC can be overly optimistic [13]. This is because the curve will remain near the top-left even if the model simply flags most samples as negative (achieving high specificity). Thus, while AUC is useful for comparing models in balanced settings or for overall discrimination ability, it should be complemented with other metrics when positive cases (e.g., a specific fungus) are rare.

***AUPRC (Area Under the Precision-Recall Curve)*:** The Precision-Recall curve plots precision against recall at various thresholds, and AUPRC is the area under this curve. Unlike AUROC, AUPRC focuses only on the performance for the positive class, ignoring true negatives. This metric is particularly informative for imbalanced datasets [13]. When positive instances are scarce (as is often the case for rare microbial species or in screening tasks), a model can achieve a high AUROC by doing well on the abundant negative class, but AUPRC will highlight performance on the minority positive class. AUPRC increases only when the model improves both precision and recall for positives. For example, in a polymicrobial detection scenario where a model looks for the presence of *Aspergillus* in plate images (a relatively rare event), the AUPRC would give a clear sense of the model’s ability to catch *Aspergillus* without raising too many false alarms. Generally, PR curves are more informative than AUROC curves for evaluating classifiers on imbalanced microbiological data [13]. AUPRC close to 1 indicates the model maintains high precision across a range of high recall values, whereas a low AUPRC indicates that as the model tries to detect more true positives, precision drops significantly (suggesting many false positives). In practice, when screening for an uncommon pathogen in a large set of images, a high AUPRC is desirable to ensure the model is reliable in finding those few positive cases.

***Confusion Matrix.*** A confusion matrix is a tabular summary of model predictions vs. true labels. For binary classification, it is a 2×2 table with cells for TP, TN, FP, and FN. For multi-class problems, it is an *N×N* matrix (for N classes) where entry (i,j) is the number of samples of true class *i* that were predicted as class *j*. The diagonal entries (i,i) represent correctly classified samples for each class [14, 15]. The confusion matrix provides a detailed view of which classes are being confused by the model. In image-based microbial ID, this is extremely useful. For example, a confusion matrix might reveal that *Streptococcus pneumoniae* colonies are often misidentified as *Streptococcus mitis* (off-diagonal entries), or that *Aspergillus fumigatus* vs *Aspergillus niger* are commonly confused. By examining the matrix, researchers can identify systematic errors, e.g., if a model confuses all yeast colonies as *Candida albicans*, indicating a possible need for more training images of other yeasts. The matrix also allows calculation of class-specific metrics: each row’s recall (sensitivity) and each column’s precision for that class. In studies, confusion matrices are frequently shown to demonstrate model performance on each organism [16]. For instance, in a deep learning classifier of four bacterial species, the confusion matrix would illustrate how many *E. coli* images were correctly identified versus mistaken for *Klebsiella* or *Enterococcus*, etc. This granular insight goes beyond a single metric, helping validate that the model’s errors make sense (e.g., confusing morphologically similar species) and guiding further improvements.

***Matthews Correlation Coefficient (MCC)*:** MCC is a single summary metric that considers all four confusion matrix outcomes (TP, TN, FP, FN) and is often regarded as a balanced measure for binary classification. It is essentially the correlation coefficient between the observed and predicted binary classifications, yielding a value between –1 and 1 [6]. An MCC of 1 indicates perfect prediction, 0 indicates random performance, and –1 indicates total disagreement (e.g., the model predicts every case incorrectly). The formula for MCC is $\frac{TP\times TN-FP\times FN}{\sqrt{\left( TP+FP \right)\left( TP+FN \right)\left( TN+FP \right)\left( TN+FN \right)}}$. MCC is particularly valuable for imbalanced datasets, as it remains high only if the model performs well on both the positive and negative classes (or all classes in general). In microbial image analysis, MCC provides a more truthful indicator under skewed class distributions than accuracy or F1 [6]. For example, consider a scenario of detecting a particular fungus in microscope images where 99% of images have no fungus. A classifier that always predicts “no fungus” would have 99% accuracy but an MCC near 0 (since it gets no true positives). MCC would flag that the model is ineffective despite high accuracy. In a recent deep learning study classifying hundreds of filamentous fungal colonies, accuracy remained moderately high even at fine taxonomic levels, but MCC dropped sharply, reflecting the model’s struggle with minority classes [6]. The authors noted MCC was a more *honest* performance measure in that highly imbalanced multi-class problem. In summary, MCC is a robust metric that is recommended for evaluating microbial classifiers, especially when classes are imbalanced or multiple classes are evaluated simultaneously, as it encapsulates both sensitivity and precision aspects in one number.

***Class Imbalance*.** Microbiological image datasets often suffer from class imbalance – some species are far more common than others. This imbalance can skew many metrics. As discussed, a model can achieve high overall accuracy by focusing on the predominant class, effectively “ignoring” rare classes [6]. Similarly, in a multi-class context, macro-averaged metrics (like macro-averaged precision/recall) treat each class equally, whereas micro-averaged metrics weight them by frequency. It is crucial to choose metrics that reflect performance on all classes. Balanced accuracy and MCC explicitly account for imbalance and give a more realistic assessment of a model’s utility on rare pathogens. Precision-Recall curves are also informative: on imbalanced data, a high AUPRC means the model is detecting rare positives with reasonable precision, whereas a high AUROC alone might be achieved by simply classifying most cases as negative. In practice, reporting class-specific precision, recall, and F1 for each organism is recommended to ensure that, for example, a deep learning model identifies *Listeria monocytogenes* (perhaps a rare class) nearly as well as it identifies *E. coli* (a common class). For instance, a recent study on fungal colony classification found that accuracy at the phylum level was 83%, but this dropped to ~49% at the order (species-group) level due to class imbalance; the corresponding MCC at order level was only ~0.21 [6], underscoring how imbalance inflated accuracy relative to a more balanced metric.

**Polymicrobial Samples:** In some images (e.g., culture plates or mixed microscopy fields), multiple species may be present simultaneously (polymicrobial samples). This poses additional challenges for evaluation. If the task is framed as multi-label classification or detection (identifying all species present in an image), the standard metrics above need adaptation. One can compute precision and recall for each organism’s detection (treating each species as a “positive” class in its own binary evaluation). For example, on a blood agar plate containing both *S. pneumoniae* and *Haemophilus influenzae*, a model might correctly detect *S. pneumoniae* but miss *H. influenzae*. In a multi-label confusion matrix, that image contributes to false negative for *H. influenzae* and a true positive for *S. pneumoniae*. The per-species recall would reflect that *H. influenzae* was missed, while per-species precision would reflect if either was falsely detected. In such cases, metrics like accuracy are less useful (since each image has more than one true label). Instead, mean average precision or per-image F1-scores (e.g., *subset accuracy* if an image is only counted correct when all species are correctly identified) can be reported. Polymicrobial identification is inherently difficult: models might have to draw multiple bounding boxes or labels. Researchers often turn to object detection metrics (like mean Average Precision at certain thresholds) for this scenario. However, if a model is only allowed to predict one label per image (single-label classification) despite polymicrobial content, its performance will inherently be limited. In such a case, the confusion matrix will show that images of a mixed culture are often misclassified as just one of the constituents. Thus, for polymicrobial plate images, careful interpretation of metrics is required. A high sensitivity for one organism might coincide with low sensitivity for another. It is important to tailor the evaluation to the task definition (single-label vs. multi-label). In summary, polymicrobial datasets demand a more nuanced set of metrics, ensuring that partial identification is quantified (e.g., using per-class recalls or multi-label F1), so that the model’s ability to handle mixed cultures is properly measured.

**References**

1.	Rainio, O., J. Teuho, and R. Klen, *Evaluation metrics and statistical tests for machine learning.* Sci Rep, 2024. **14**(1): p. 6086.

2.	Hospodsky, D., N. Yamamoto, and J. Peccia, *Accuracy, precision, and method detection limits of quantitative PCR for airborne bacteria and fungi.* Appl Environ Microbiol, 2010. **76**(21): p. 7004-12.

3.	Subramanian, M., et al., *Learning without forgetting by leveraging transfer learning for detecting COVID-19 infection from CT images.* Sci Rep, 2023. **13**(1): p. 8516.

4.	Thölke, P., et al., *Class imbalance should not throw you off balance: Choosing the right classifiers and performance metrics for brain decoding with imbalanced data.* NeuroImage, 2023. **277**: p. 120253.

5.	Albattah, W. and R.U. Khan, *Impact of imbalanced features on large datasets.* Front Big Data, 2025. **8**: p. 1455442.

6.	Stiller, S., et al., *Deep learning image analysis for filamentous fungi taxonomic classification: Dealing with small datasets with class imbalance and hierarchical grouping.* Biol Methods Protoc, 2024. **9**(1): p. bpae063.

7.	Majchrowska, S., et al., *AGAR a microbial colony dataset for deep learning detection.* arXiv preprint arXiv:2108.01234, 2021.

8.	Burns, B.L., D.D. Rhoads, and A. Misra, *The Use of Machine Learning for Image Analysis Artificial Intelligence in Clinical Microbiology.* J Clin Microbiol, 2023. **61**(9): p. e0233621.

9.	Cicatka, M., et al., *Increasing segmentation performance with synthetic agar plate images.* Heliyon, 2024. **10**(3): p. e25714.

10.	Guesne, S.J.J., et al., *Mind your prevalence!* J Cheminform, 2024. **16**(1): p. 43.

11.	Couto da Silva, C.M., *A Data Scientist’s Guide to Balanced Accuracy*. 2024.

12.	Nanny, M.L. *Creating and Estimating a Custom Binary Classification Metric*. 2022 2022 [cited 2026 06.01.2026]; Tutorial]. Available from: <https://nannyml.readthedocs.io/en/main/tutorials/performance_estimation/binary_performance_estimation/custom_metric_estimation.html#:~:text=As%20a%20reminder%2C%20the%20balanced,accuracy%20is%20defined%20as>.

13.	Saito, T. and M. Rehmsmeier, *The precision-recall plot is more informative than the ROC plot when evaluating binary classifiers on imbalanced datasets.* PLoS One, 2015. **10**(3): p. e0118432.

14.	Signoroni, A., et al., *Hierarchical AI enables global interpretation of culture plates in the era of digital microbiology.* Nature Communications, 2023. **14**(1): p. 6874.

15.	Jacot, D., et al., *Development and evaluation of an artificial intelligence for bacterial growth monitoring in clinical bacteriology.* J Clin Microbiol, 2024. **62**(5): p. e0165123.

16.	Subramanian, M., et al., *Learning without forgetting by leveraging transfer learning for detecting COVID-19 infection from CT images.* Scientific Reports, 2023. **13**(1): p. 8516.
